# An ultrasound and shear wave elastography study: effect of grip on medial elbow joint morphology during valgus stress

**DOI:** 10.1186/s12891-025-08343-2

**Published:** 2025-02-26

**Authors:** Matthew F. Gong, Tyler J. Wilps, Jamieson G. Abrams, Shanelle Dorn, Jesal N. Parekh, Tudor H. Hughes, Catherine M. Robertson, Matthew J. Meunier, Samuel R. Ward

**Affiliations:** 1 Department of Orthopedic Surgery, Muscle Physiology Lab, ACTRI, University of California San Diego, 9500 Gilman Dr. #0863, La Jolla, San Diego, CA 92093 USA; 2 Department of Radiology, Muscle Physiology Lab, ACTRI University of California San Diego, 9500 Gilman Dr. #0863, La Jolla, San Diego, CA 92093 USA

**Keywords:** Elbow ultrasound, Ulnar collateral ligament, Forearm flexor-pronator mass, Injury prevention

## Abstract

**Background:**

The flexor pronator mass (FPM) is an important dynamic stabilizer to valgus stress at the elbow and has been reported to protect against ulnar collateral ligament (UCL) injury. Active gripping and pronation have demonstrated reduced ulnohumeral joint space and change in material properties of the UCL when examined in vivo via ultrasound. No studies have utilized ultrasonography and shear wave elastography to characterize the medial elbow’s response to FPM activation under valgus stress. This cross-sectional, repeated measures study aims to characterize medial elbow changes in UCL and FPM tissue stiffness and joint space width (JSW) during valgus stress with incremental FPM activation through gripping.

**Methods:**

Thirteen participants (6 male, 7 female) aged 18–40 year with a BMI < 30 and no history of upper extremity injury were included in this study. Elbows were placed in a telos stress device at 30° of flexion and a 100N valgus stress was applied. Participants then activated the FPM by gripping a spherical dynamometer at 100, 75, 50, 25, and 0% of maximal grip strength. UCL thickness, ulnohumeral (UH) JSW, UCL stiffness, and FPM stiffness were measured for each condition and compared via a two-way repeated measures ANOVA and a post hoc Fischer’s Least Significant Difference test.

**Results:**

Men and women showed no baseline differences in UCL thickness or UH JSW. JSW was significantly wider under valgus load, 2.22 ± 0.42 mm vs. 2.99 ± 0.46 mm in males and 2.15 ± 0.41 mm vs. 2.99 ± 0.55 mm in females (*p* < 0.05). No statistically significant differences were demonstrated in UH JSW by gripping force magnitude and differences by sex were not observed. Additionally, no significant changes in tissue stiffness were observed during dynamic conditions for shear wave velocities for either the UCL or FPM.

**Conclusion:**

Gripping does not change UH JSW or medial elbow tissue stiffness in the joint testing configuration and external loading conditions applied in this study. This suggests that gripping may not be as protective during the high valgus stress observed in baseball pitching as believed, and that the influence of FPM activity may be joint position or load dependent.

**Supplementary Information:**

The online version contains supplementary material available at 10.1186/s12891-025-08343-2.

## Introduction

Overhead throwing athletes are at risk for ulnar collateral ligament (UCL) injury due to repetitive valgus stress imparted to the elbow. This is best exemplified in baseball pitchers who experience frequent and significant UCL injuries which often require surgical intervention [1, 2]. The incidence of UCL reconstructions performed in the United States continues to rise, with the most concerning increase observed in the 15–19-year-old pitcher population [3–5]. As such, there is tremendous interest in understanding the mechanism of UCL injuries, associated risk factors, and strategies to protect the ligament.

The anterior band of the anterior bundle of the UCL is the primary stabilizer to valgus stress, and the flexor pronator mass (FPM) has been identified as a dynamic stabilizer that attenuates valgus stress directly via an opposing varus torque [6, 7]. Prior cadaveric studies have characterized both the flexor carpi ulnaris (FCU) and flexor digitorum superficialis (FDS) as important dynamic stabilizers whereas the pronator teres (PT) has been considered secondary [8, 9]. Players with UCL injuries who suffer a concomitant FPM injury have also been found to have worse clinical outcomes than players with UCL injuries alone [10, 11]. EMG studies further support the protective effect of the FPM by demonstrating a rapidly increasing activation of the FCU from 41% in the late cocking phase to greater than 100% activation during the acceleration phase of pitching, where elbow valgus loading is highest [12].

Ultrasound is increasingly used to monitor the medial elbow of pitchers. Ciccotti et al. measured ulnohumeral (UH) joint space width (JSW), UCL thickness, and echogenic abnormalities in professional pitchers and demonstrated that players with increased echogenic abnormalities and increased joint space at baseline were more likely to sustain UCL injury [13]. Laboratory studies have suggested that active gripping or pronation can reduce the UH JSW in vivo and affect the material properties of the UCL [14–16].

Shearwave elastography (SWE) is a non-invasive method of assessing the elasticity of tissue and has been shown to have better repeatability than strained-based techniques; however, no study has utilized shear wave elastography to assess the material properties of the medial elbow during valgus stress and incremental gripping [17]. Additionally, no study has assessed potential sex-related differences in these properties. Finally, there is no consensus on the best testing position or magnitude of valgus stress to induce medial joint space width (JSW) opening.

The purpose of this study was to characterize FPM stabilization of the medial elbow in vivo*.* We hypothesized that gripping would decrease the UH JSW despite application of valgus stress and that the change would be proportional to grip magnitude. We also hypothesized that shear wave velocity, a proxy for tissue stiffness, would increase in the UCL during valgus stress but decrease during active gripping while tissue stiffness in the FPM would increase during active gripping.

## Materials and methods

### Subject population

The study was conducted at University of California San Diego (La Jolla, CA) from 7/2018 to 7/2019, under an IRB approved protocol in accordance with the Declaration of Helsinki. Participants were recruited via open-invitation flyers and consented to participation. Inclusion required an age 18–40 years old, BMI < 30, and the ability to perform a full-strength gripping motion and tolerate ultrasound procedure, including valgus stress. Participants were excluded if they had any history of upper extremity injury, if elbow abnormalities were detected during ultrasound examination, or if they were unable to tolerate the procedure. Overhead athletes and throwers were not specifically recruited. A total of 17 subjects were recruited for the study, however, 3 subjects were excluded due to poor quality imaging studies, while 1 subject did not complete the full study protocol due to elbow soreness incurred during the ultrasound procedure. In total, 13 subjects (6 male, 7 female) completed the full ultrasound procedure (Table 1). University of California San Diego Institutional Review Board approved the study design, consent and recruitment procedure. Clinical trial number: not applicable. This is an observational experiment in humans, not a clinical trial. Every human subject provided their consent prior to undergoing the experimental ultrasound procedure.

**Table 1:** Demographic characteristics of study population

Gender	Height (cm)	Weight (kg)	BMI	Forearm Length (cm)	Grip Strength (kg)	Dominant Hand
Male (*n* = 6)	23.5 ± 1.0	52 ± 18	2.22 ± 0.42	1.40 ± 0.31	23.74 ± 8.18	100% (6) right
Female (*n* = 7)	24.1 ± 5.3	34 ± 5	2.15 ± 0.41	1.21 ± 0.19	15.49 ± 2.26	100% (7) right

### Study protocol

**Fig. 1:**
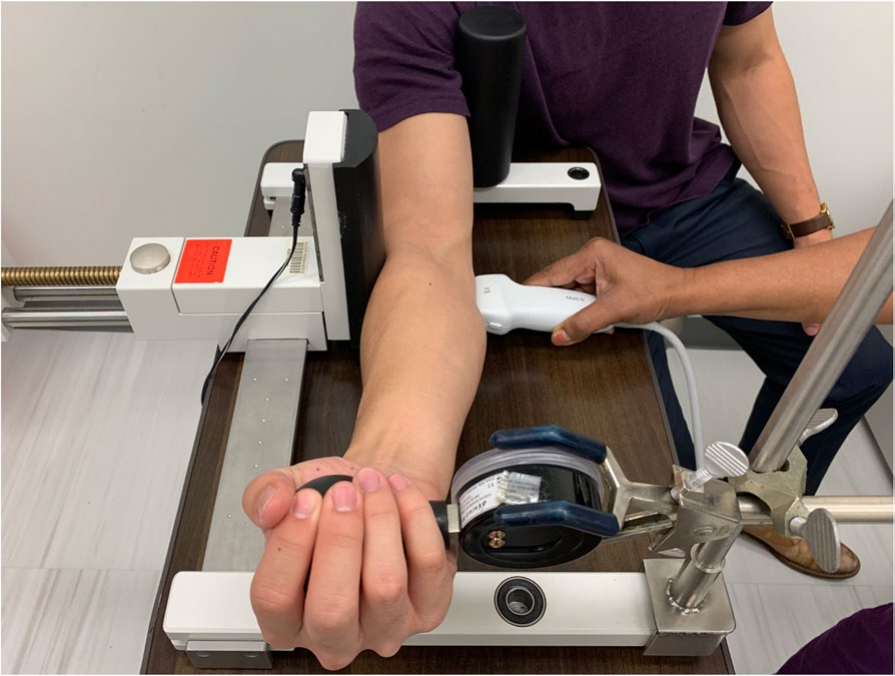
Telos stress device with modified spherical dynamometer handgrip set-up to simulate gripping a baseball. Subject's elbow is positioned at 30 degrees of flexion and a controlled valgus load is applied through the device

This study was a prospective, basic science study comparing ultrasound-based measurements in individuals under valgus conditions during periods of dynamic muscle gripping. A standardized valgus force was applied utilizing a modified Telos Stress Device similar to the protocol implemented by Ciccotti et al [13]. The subject’s arm was positioned in 30º elbow flexion while supinated, and a 100N valgus force was applied, rather than the previously described 150N, to limit participant discomfort [13]. A Saehan Squeeze Dynamometer was fitted as a handgrip and served to assess grip strength while mimicking the action of gripping a baseball (Fig. 1). Maximal or 100% gripping was determined by an average of three trials where subjects were encouraged to squeeze the dynamometer with maximal strength, and target outputs were determined from this average prior to positioning in the Telos device. This experimental set-up allowed for reproducible joint position and loading across the elbow. Images of the medial elbow were acquired under 8 distinct conditions: 1) Baseline unloaded, 2) Pre-exertion valgus, 3) Valgus with 100% gripping, 4) Valgus with 75% gripping, 5) Valgus with 50% gripping, 6) Valgus with 25% gripping, 7) Post-exertion valgus, and 8) Post-loading unloaded.

### Ultrasound imaging technique

**Fig. 2:**
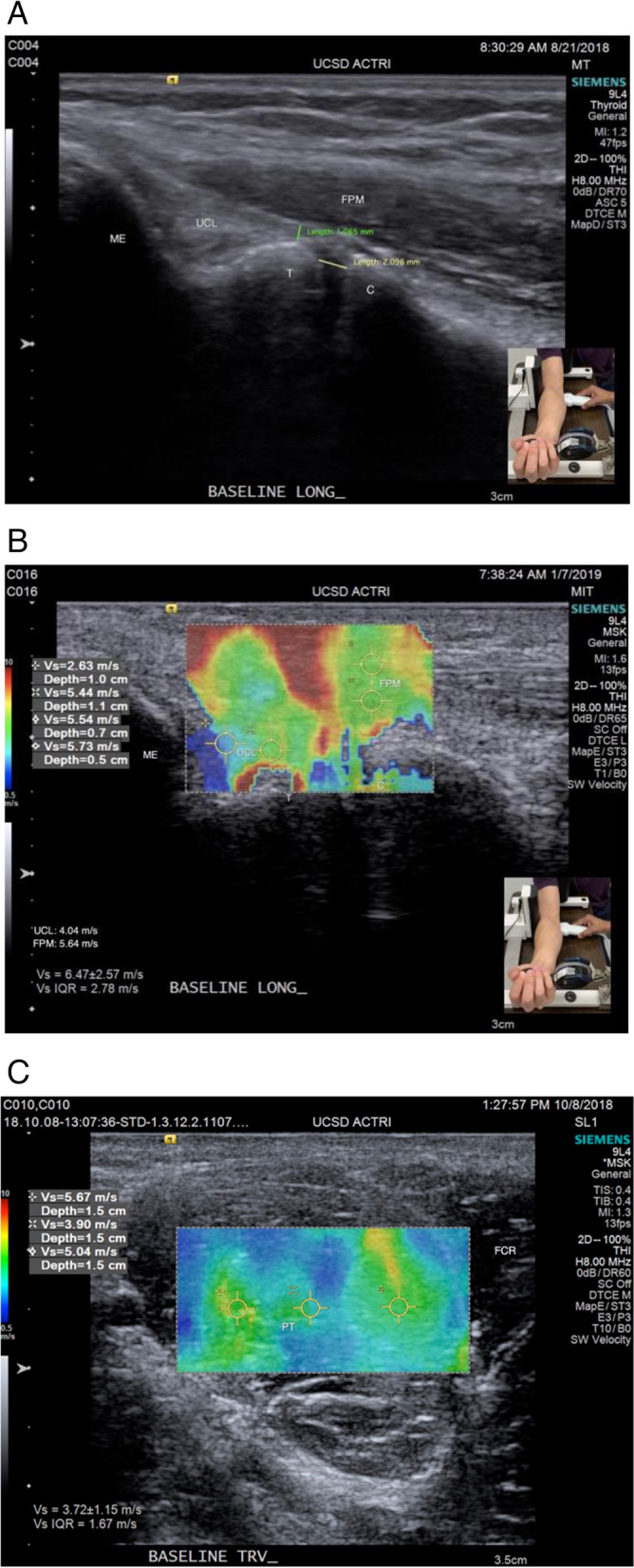
**a**: Baseline longitudinal ulnar collateral ligament (UCL) ultrasound image with reference measurements for UCL thickness and ulnohumeral joint space width (JSW). Ultrasound probe positioning as shown in Fig. 1. **b**: Baseline longitudinal UCL shear wave elastography image with demonstration of sampling method of shear wave velocity for UCL and for the flexor pronator mass. Longitudinal measurements comprised the region of flexor pronator mass muscle overlying the UCL. Ultrasound probe positioning as shown in Fig. 1. **c**: Baseline transverse pronator teres (PT) elastography image with demonstration of sampling method of muscle shear wave velocity. Measurements were taken in the transverse PT muscle body. Acuson SC3000 with a velocity range set at 0.5 to 10 m/s. Ultrasound robe positioning is orthogonal to that shown in Fig. 1

Four types of images were acquired using a Siemens Acuson SC3000 using a 9L4 linear array transducer operating at 8 MHz: 1. Longitudinal b-mode images of the UCL (Fig. 2a), 2. Longitudinal SWE images of UCL (Fig. 2b), 3. Transverse b-mode images of the FPM, 4. Transverse SWE images of the FPM (Fig. 2c). These sets of ultrasound images were taken by the same certified ultrasound technician. To assess repeatability of ultrasound measurements, we calculated the Coefficient of Variation (CV) for UCL thickness and JSW using the standard deviation of the absolute differences between two repeated measurements calculated for each subject divided by the mean of the original measurements. This method accounts for the actual size of measurements. CV was 11.83% for UCL thickness and 14.48% JSW.

### Imaging measurements

All images were imported to Horos 3.3.0 for digital measurements. UCL thickness was measured at the most medial apex of the trochlear cortex perpendicular to the course of the UCL (Fig. 2a). Ulnohumeral joint space width was measured via a line parallel and 1 mm deep to the deep border of the UCL’s fibers, with the measured joint space spanning the intersection of this line from the distal humeral cortex to the proximal ulnar cortex.

Shear-wave elastography measurements were taken from the shear wave velocity (SWV) recordings sampled from the 2 tissues of interest, the UCL and the FPM. For the FPM SWV measurements, the longitudinal measurements comprised the region of FPM muscle overlying the UCL, whereas the transverse measurements were taken in the transverse PT muscle body due to the superficial position and the inability to capture SWV measurements consistently in deeper muscles of the FPM. The SWV measurements were measured on the Acuson SC3000 with a velocity range set at 0.5 to 10 m/s. Two and three regions were sampled and averaged to determine SWV for the UCL and FPM, respectively.

### Statistical analysis

A 2-way repeated measures ANOVA (alpha = 0.05) with multiple comparisons analyses were conducted to assess for interactions between sex and between each of the 8 experimental conditions. A post hoc Fisher’s Least Significant Difference Test was then conducted to determine specific pairwise differences between groups and conditions. A priori power calculations based on joint space width differences between no-load and valgus load conditions from Pexa (Cohen’s D = 0.712), alpha = 0.05, and beta = 0.95) indicated that a minimum of 12 total subjects would be required for this repeated measures design [16, 18].

## Results

### UCL Thickness

The mean UCL thickness measured at baseline was 1.40 ± 0.31 mm in male subjects, 1.21 ± 0.19 mm in female subjects, and 1.30 ± 0.26 overall. (Table 2). UCL thickness did not change in a statistically significant manner when valgus stress was applied nor in any dynamic gripping conditions.

**Table 2:** Baseline data of study population

Gender	UCL thickness (mm)	Joint Space Width (cm)	UCL SWE (m/s)	FPM Longitudinal SWE (m/s)	FPM Transverse SWE (m/s)
Male (*n* = 6)	1.40 ± 0.31	2.22 ± 0.42	5.18 ± 1.25	4.43 ± 1.64	3.70 ± 1.10
Female (*n* = 7)	1.21 ± 0.19	2.15 ± 0.41	4.68 ± 1.97	6.1*3* ± *1.85*	3.41 ± 1.58

### Ulnohumeral joint space width

**Fig. 3:**
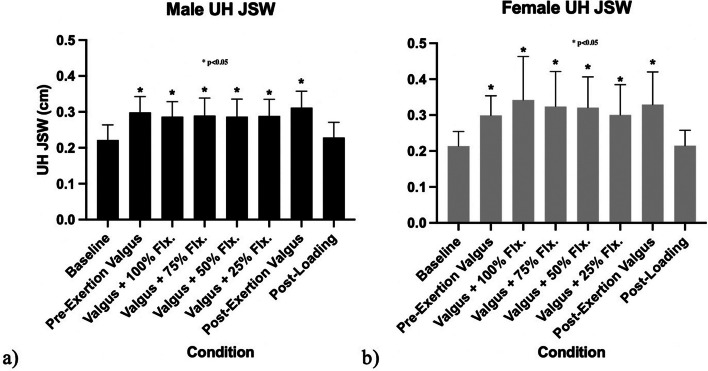
Comparison between **a**) male and **b**) female changes to ulnohumeral joint space over progression of ultrasound study, with condition described along x-axis. *p < 0.05 denotes a statistically significant difference from the baseline condition under repeated measures ANOVA and post hoc Fisher’s Least Significant Difference. When compared to pre-exertion valgus or post-exertion valgus conditions, there were statistically significant differences in the conditions involving all conditions with valgus loading regardless of FPM contraction

The mean UH joint space width measured at baseline was 2.22 ± 0.42 in male subjects and 2.15 ± 0.41 mm in female subjects (Table 2). The UH joint space width increased in both men and women during all valgus stress conditions regardless of the magnitude of gripping when compared to baseline (*p* < 0.05, Fig. 3). No gripping condition significantly reduced the joint space width. The greatest joint space widths were recorded during the post exertion valgus condition and were 3.11 ± 0.46 mm in males and 3.29 ± 0.90 mm in females. (Fig. 3).

### Shear wave velocity

**Fig. 4:**
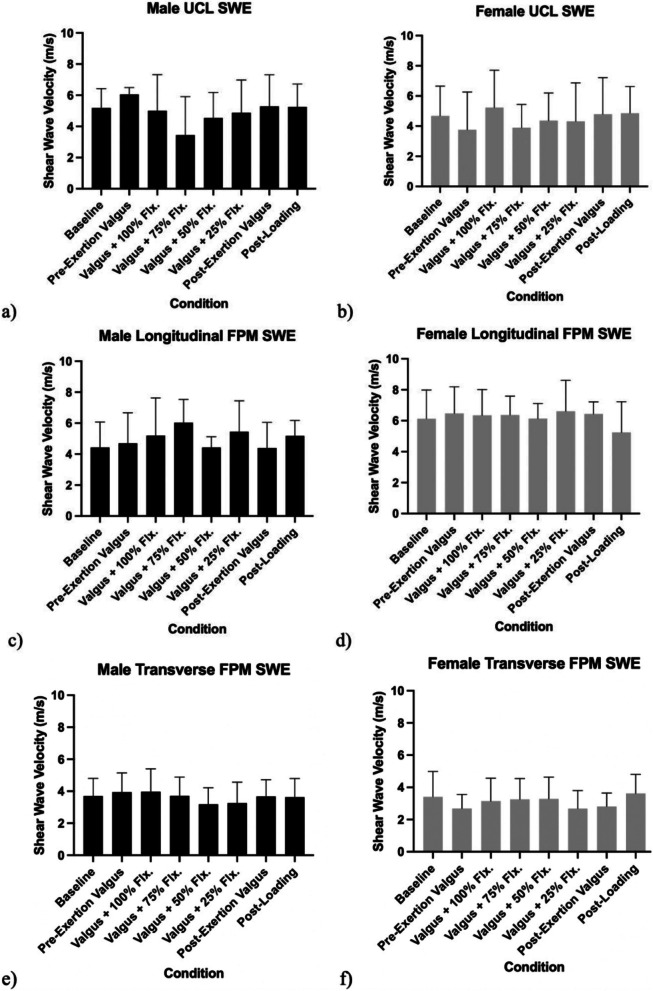
Shear wave velocity (SWV) measurements of tissue stiffness in the ulnar collateral ligament for a) male and b) female subjects. Mean baseline SWV for UCL in males was 5.18 ± 1.25 m/s and in females was 4.68 ± 1.97 m/s. SWV measurements in the longitudinal flexor pronator mass for c) male and d) female subjects. Mean baseline SWV for longitudinal FPM in males was 4.43 ± 1.64 m/s and in females was 6.13 ± 1.85 m/s. SWV measurements of tissue stiffness in the transverse flexor pronator mass for e) male and f) female subjects. Mean baseline SWV for transverse FPM in males was 3.70 ± 1.10 m/s and in females was 3.41 ± 1.58 m/s

The mean baseline shear wave velocity (SWV) measurements in males were 5.18 ± 1.25 m/s for the long-axis (LA) UCL, 4.43 ± 1.64 m/s for the LA FPM, and 3.70 ± 1.10 m/s for the transverse FPM. In females, the measurements were 4.68 ± 1.97 m/s for the LA UCL, 6.11 ± 2.02 m/s for the LA FPM, and 3.41 ± 1.58 m/s for the transverse FPM (Table 2). SWV measurements of the LA UCL, LA FPM, and transverse FPM did not demonstrate any statistically significant change over the course of the experiment under valgus stress or dynamic gripping conditions. (Fig. 4).

## Discussion

Our results suggest that active gripping does not influence medial elbow joint space width or tissue stiffness during valgus stress. Our study also did not detect any sex-associated differences in medial elbow morphology or response to valgus stress and FPM activation. Ultrasound, as an imaging modality, offers several advantages, including its temporal and non-invasive nature, ease of use, low cost, and absence of ionizing radiation. In our study we utilized both B-mode imaging to measure UCL thickness and ulnohumeral JSW as well as shear wave elastography to measure tissue stiffness.

The coefficient of variation (CV) between repeated ultrasound UCL thickness and JSW measurements at two different time points was 11.8% and 14.5%, respectively, indicating acceptable repeatability. The average absolute difference between repeated measures for JSW was 0.34 mm, which is smaller than the recorded change in JSW throughout our study (0.89 mm in men and 1.14 mm in women). This suggests that the experimental design induced a change in JSW that was greater than the inherent measurement variability (system noise).

### UCL Thickness

As expected, our study noted UCL thickness to be less than what has been reported in professional baseball players, 6.15 ± 1.57mm [13]. Thickening of the UCL ligament is an adaptive response known to occur in throwing athletes, and these measurements contextualize UCL thickness in a non-throwing cohort [13, 19]. No consensus for measuring UCL thickness exists in the literature with varying techniques including the midportion of the UCL anterior bundle from the superficial ligament to cortical bone [19, 20], at the midportion of only the superficial ligament [21, 22] or at proximal, medial, and distal thirds of the ligament [23].

Greater UCL thickness may be obtained by extending measurements to cortical bone and by measuring more proximally where the ligament is generally thicker at baseline and in response to pitching. In our study, the most medial apex of the trochlea was a clear identifiable sonographic landmark ideal for making a consistent measurement; however, thickening may be more evident if measured proximally. Further study is required to determine where UCL thickness measurements should be made for the highest reproducibility and clinical utility.

### Joint space

Our findings are in contrast with prior in-vivo ultrasound studies that have demonstrated decreased JSW with FPM activation and may be accounted for, in part, by differences in experimental set up [14–16]. Hattori et al. conducted a dynamic ultrasound elastography study in 29 healthy college students at 90 degrees of elbow flexion and applied a 4 kg weight to the forearm in the unloaded position to maintain 90 degrees of external rotation of the shoulder on the examination table. They observed a statistically significant difference in UH JSW at maximal grip strength (3.1 ± 0.6 mm) compared to rest (3.8 ± 0.8 mm) [14]. As little as a 2.4 kg valgus stress has been reported to show difference in UH JSW in more recent ultrasound studies [24].

Our experimental set up employed a significantly higher 100N (> 10 kg) valgus load at 30 degrees of elbow flexion, similar to the protocol defined by Ciccotti et al.; however, the valgus force was reduced from 150 to 100N to limit participant discomfort. The 100N valgus load effectively increased medial elbow JSW by 0.89 mm (40%) and 1.14 mm (53%) in males and females, respectively; whereas a 150N valgus load increased medial JSW by 1.24 mm (37.3%) in the dominant arm of professional pitchers [13].

It is possible that the FPM contraction was not sufficient to overcome the applied valgus load in our study. Indeed, average maximum grip strength in our cohort was 19.41 ± 6.72 kg compared to a nearly twofold stronger grip strength reported in the dominant arm of high school pitchers, 37.8 ± 5.7kg [25]. Consideration may be given to optimizing the valgus force applied based on patient population and grip strength. Additionally, whole hand gripping in a neutral to extended forearm position may activate both the FDS and the extensor mass resulting in unreliable protective varus torque. Otoshi et al. found that pronation was the most effective wrist motion to reduce UH JSW during an in-vivo ultrasound experiment [15].

The commonly used telos device applies reliable valgus loads but due to the design, requires measurements at less than 60° elbow flexion. The UCL is most important in valgus stability of the elbow at 30° elbow flexion, but stress imaging of the elbow by ultrasound and MRI is becoming increasingly performed in 90° of elbow flexion to mimic arm position at late cocking and acceleration phase of the pitching cycle [26–28].

Comparably, Pexa et al. compared joint space width in twenty-two non-pitchers at 30° of elbow flexion during valgus stress at rest and maximal grip contraction (4.91 ± 1.16 vs 4.26 ± 1.23 mm) [16]. Chalmers et al. found increased UH JSW in professional baseball pitchers after a season of play at 30° but no change at 90° elbow flexion and speculated that pitchers are better able to muscularly compensate for valgus stress at 90° flexion [24]. Kissenberth et al. also examined the elbow in 90 degrees of flexion and reported thicker UCL’s in professional pitchers with prior UCL reconstruction than non-injured players (1.1 ± 0.08 mm vs 0.17 ± 0.07), and the reconstruction group demonstrated less joint space gapping under a 2.5 kg valgus load 4.7 ± 0.86 mm vs 5.4 ± 1.2 [29].

Importantly, the act of pitching involves dynamic wrist motion during the late cocking and acceleration phase. Peak wrist angles during the cocking phase have been reported at 41° wrist extension, 15° radial deviation, and 17° forearm supination [30]. As maximal external rotation of the shoulder is achieved, and the arm acceleration phase begins, peak angular velocities at the wrist have been reported at 2,954°/s wrist flexion and 2,055°/s ulnar deviation with forearm pronation occurring early in the acceleration phase [30]. The wrist flexion moment in combination with ulnar deviation and pronation occurring during maximal elbow valgus torques correlates with reported EMG activity of FCU and FCR greater than 100% during the acceleration phase, whereas FDS EMG activity averaged 80% [12]. The complexity of dynamic stability during pitching, including active muscle shortening at the wrist, may call into question the utility of studying the effect of isometric FPM activity on elbow valgus because FPM load magnitude will be lower during active shortening than during the isometric conditions utilized in experimental studies.

Though specific muscle activation of FPM may dynamically protect the UCL against valgus stress, there is the possibility that the protective effect is less pronounced than previously suggested. Several mechanisms may account for the discrepancy between *in-vitro* valgus torque maximums and those observed during baseball pitching (40-120Nm) [31, 32]. The radial-capitellar joint as well as the posteromedial olecranon provide a bony buttress to resist valgus torque [27]. Other muscles such as the biceps and triceps have been shown to reduce varus/valgus laxity through secondary joint compression effects [33]. Triceps activation, in particular, has been shown to occur before maximal external rotation and may contribute to dynamic stability against valgus stress [12].

### Shearwave elastography

Shear wave elastography was also piloted as a tool for studying the UCL and FPM under valgus stress and dynamic gripping, and our results elicited no statistically significant findings. Reproducibility of SWV in actively contracted muscle was challenging, and similar challenges were reported in a study by Gupta et al., which measured SWV of twenty elbows in ten healthy volunteers. Despite these challenges, mean UCL SWV in our study was comparable to the 5.14 m/s measured from the 10 dominant elbows imaged in the aforementioned study [17].

Increased ligament stiffness as an adaptive responsive has been previously reported, including a study using the Acuson ultrasound where increased transverse carpal ligament stiffness was observed in pianists (5.52 m/s) compared to non-pianists (5.01 m/s) [34]. Shear wave velocity measurements of the supraspinatus muscle have also demonstrated increased stiffness when comparing actively contracting muscle (3.74 m/s) to passively resting muscle (2.23 m/s) [35].

Other studies have also validated that skeletal muscle subjected to an increased tensile load will have measurably increased stiffness [36]. Most recently, Hattori et al. demonstrated that elastography measurements of the UCL and FPM can be measured with excellent reliability and observed that maximal grip strength reduced elasticity of both the UCL and FPM when compared to the resting state [14]. Of note, our study employed transverse SWV measurements in the body of the PT muscle which may not be preferentially activated during whole hand gripping.

This study has several limitations. Although a priori power calculation indicated a minimum requirement of 12 total subjects, our relatively small sample size may limit the study's ability to detect true differences, increasing the risk of a Type II error. Additionally, the population of non-overhead athletes and testing position, which does not perfectly replicate the valgus stress of pitching, limit the applicability of results to the most at risk population for medial elbow injuries. However, this position is better suited for standardized loading in a test frame. Furthermore, shear wave elastography has not been shown to be a very repeatable measurement when assessing medial elbow structures.

## Conclusion

In our study, active gripping does not reliably reduce ulnohumeral joint space or UCL stiffness when a 100N valgus load is applied in non-overhead throwing athletes in 30 degrees of elbow flexion. The medial elbow response to valgus load, with and without FPM activation, is also not significantly different between male and female non-overhead athletes. These data, along with a synthesis of the literature, call into question the relative influence of testing configurations (valgus load, elbow position, forearm position, grip load, and grip position). As a result, the utility of using a static measurement tool to study the protective effects of FPM strength and endurance on the UCL during pitching remains unclear. Additional in vivo work on the relationship between experimental conditions and UCL biomechanics is warranted, and longitudinal studies on the relationship between FPM training and reduced UCL injuries is necessary.

## Supplementary Information

Supplementary Material 1.

## Data Availability

Raw data for this dynamic ultrasound study is available for viewing in Appendix I. Ultrasonic images are presented in the context of the research article but are not provided in full to protect patient health information. For any questions, please contact the lead author, Matthew F Gong and Samuel R Ward.
